# Supplementary material for: Niacin restriction with NAMPT-inhibition is synthetic lethal to neuroendocrine carcinoma
Source: Nat Commun. 2023 Dec 13;14:8095. doi: 10.1038/s41467-023-43630-3 (PMC10719245; doi:10.1038/s41467-023-43630-3)
Supplement: Supplementary file 1 — Supplementary Information [file 41467_2023_43630_MOESM1_ESM.pdf]

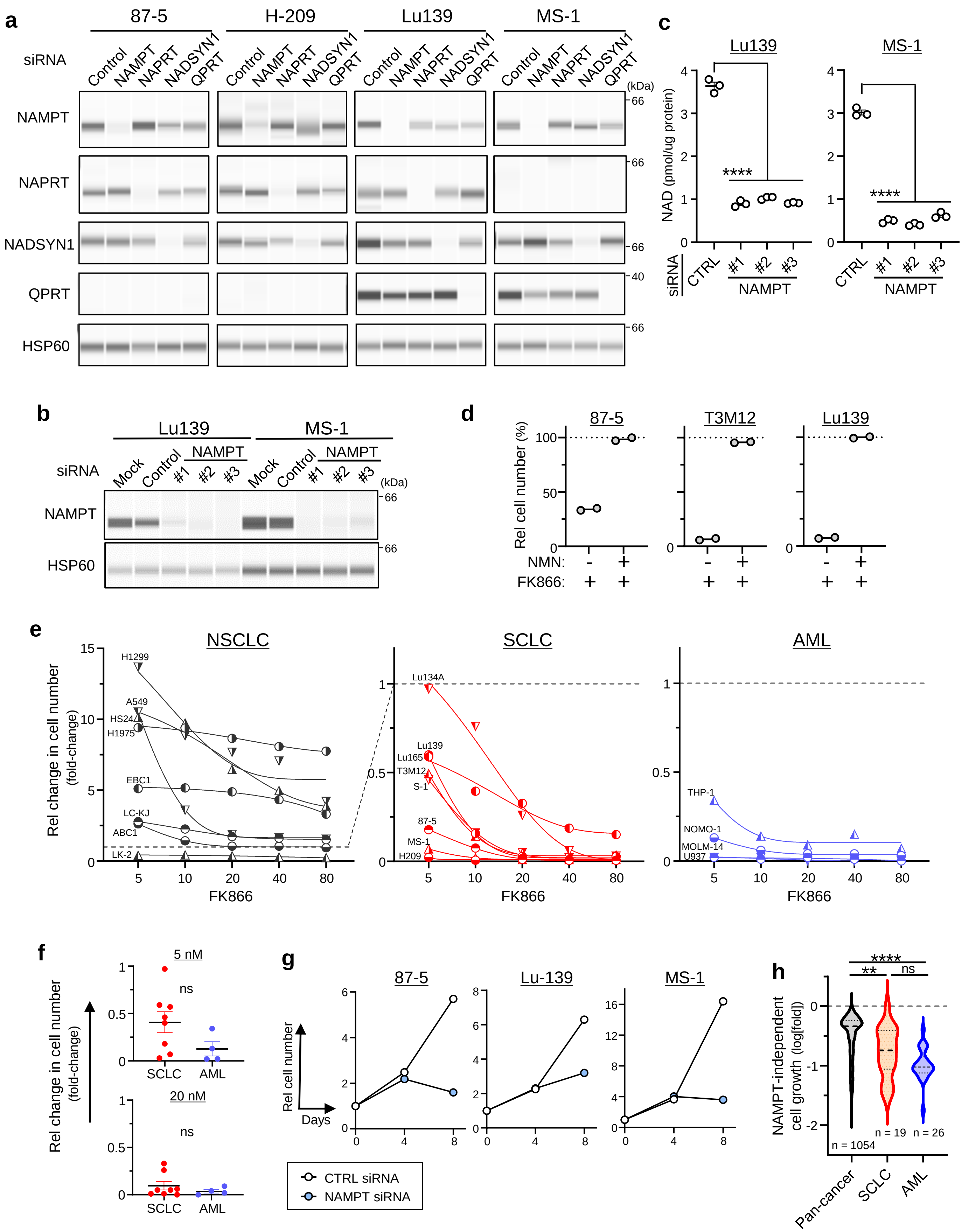
a
87-5
H-209
Lu139
MS-1
siRNA
Control
NAMPT
NAPRT
NADSYN1
QPRT
HSP60
(kDa)
66
40
b
Lu139
MS-1
siRNA
Mock
Control
NAMPT
#1
#2
#3
c
Lu139
MS-1
NAD (pmol/ug protein)
****
CTRL
d
87-5
T3M12
Rel cell number (%)
NMN:
FK866:
e
NSCLC
SCLC
AML
Rel change in cell number
(fold-change)
H1299
A549
HS24
H1975
EBC1
LC-KJ
ABC1
LK-2
Lu134A
Lu165
T3M12
S-1
MS-1
H209
THP-1
NOMO-1
MOLM-14
U937
FK866
f
5 nM
20 nM
ns
SCLC
AML
g
Lu-139
Rel cell number
Days
CTRL siRNA
NAMPT siRNA
h
NAMPT-independent
cell growth (log[fold])
**
ns
n = 1054
n = 19
n = 26
Pan-cancer

Supplementary Fig. 1. NAMPT is essential for SCLC cell survival/proliferation, related to Fig. 2.
(**a**) Immunoassays showing knock-down efficiency of indicated siRNAs in transiently-transfected SCLC cells. Note that NAPRT in MS-1 cells and QPRT in 87-5 and H209 cells were under the protein detection limits. Shown is a representative of >3 independent experiments.
(**b**) Immunoassays showing knock-down efficiency of 3 types of NAMPT siRNAs in transiently-transfected SCLC cells. NAMPT siRNA #1 is the same as that used in **a**. Shown is a representative of >2 independent experiments.
(**c**) NAD levels in cells treated as in **b**. n = 3 biological replicates. $p < 0.0001$ (CTRL vs #1, Lu-139); $p < 0.0001$ (CTRL vs #2, Lu-139); $p < 0.0001$ (CTRL vs #3, Lu139); $p < 0.0001$ (CTRL vs #1, MS-1); $p < 0.0001$ (CTRL vs #2, MS-1); $p < 0.0001$ (CTRL vs #3, MS-1).
(d) Effects of exogenous NMN (a metabolite produced by NAMPT in the NAD salvage pathway) on growth suppression by FK866. Cells were treated 4 days with 20 nM FK866 with or without 1 mM NMN. Results are shown as relative to non-treated cells, which were defined as 100%. n = 2 biological replicates.
(e) Dose response curves of SCLC, NSCLC and AML cell lines to FK866. Cells were treated 4 days with 5-80 nM FK866. Shown are relative numbers of viable cells compared to those at day 0, defined as 1.0 (denoted as dashed line), and are representative of two independent experiments.
(f) Results in **e** were compared between SCLC and AML groups. $p = 0.1257$ (5 nM); $p = 0.3933$ (20 nM).
(**g**) Effects of siRNA-mediated NAMPT knockdown on SCLC line proliferation. siRNA transfection was performed twice (on days 0 and 4). Data are representative of two independent experiments.
(**h**) Analysis of the DepMap dataset showing effects of NAMPT knock-out on proliferation of cell lines in the CCLE collection. Results were compared in pan-cancer, SCLC and AML categories. $p = 0.0017$ (pan-cancer vs SCLC); $p < 0.0001$ (pan-cancer vs AML); $p = 0.1683$ (SCLC vs AML).
Data are presented as the mean of measurement duplicates (**e**, **g**), the mean of biological replicates (**d**), or the mean of biological replicates plus SEM (**c**, **f**, **h**). *p < 0.05, ****p < 0.0001 as determined by one-way ANOVA with a post-hoc test (**f** and **i**) or by two-tailed t-test (**g**). ns., not significant. Source data are provided as a Source Data file.

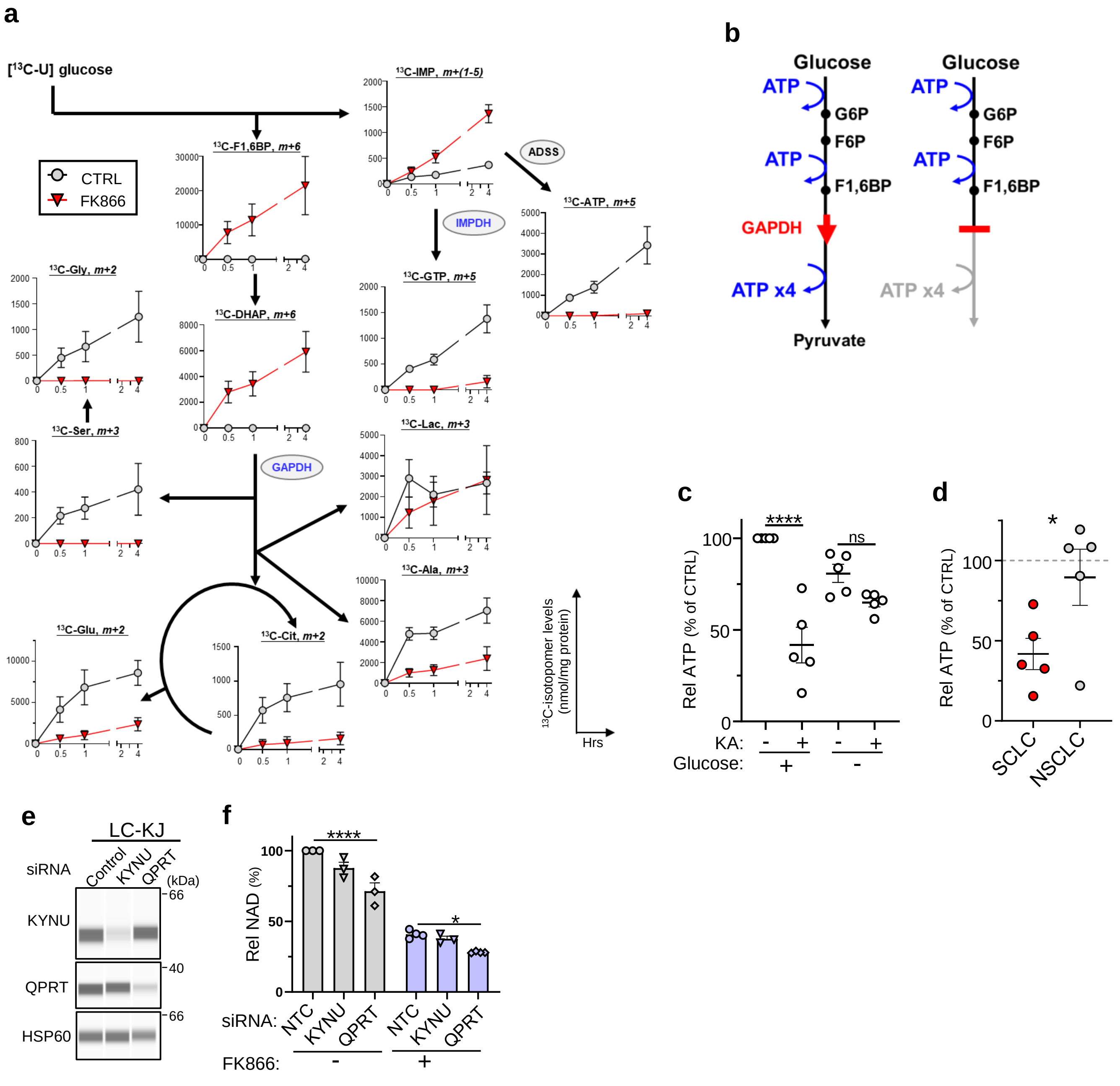

Supplementary Fig. 2. NAMPT inhibition disrupts SCLC cell bioenergetics, related to Figs. 2 and 3.

(a) Time course analysis using [U-^{13}C] glucose as a tracer in 5 SCLC cell lines (87-5, H209, Lu-139, MS-1, and T3M-12). Accumulation of ^{13}C-glucose-derived isotopomers of each metabolite is shown along with metabolic pathways. Results are shown as averages of the 5 cell lines.

(b) Consequences of GAPDH inactivation. In glycolysis, metabolizing one molecule of glucose consumes 2 ATPs and produces 4 ATPs upstream and downstream of GAPDH, respectively. Thus, GAPDH inhibition converts glycolysis from an energy-producing to an energy-consuming pathway.

(c) ATP levels in 5 SCLC lines treated 6 hrs with either the GAPDH inhibitor Koningic acid (KA) or control vehicle, in the presence or absence of glucose. Each symbol shows representative results of the 5 SCLC lines in **a**. p < 0.0001 (wo KA vs w/ KA, w/ glucose); p = 0.2244 (wo KA vs w/ KA, wo glucose).

(d) ATP levels in cells of 5 NSCLC and 5 SCLC lines after 6 hrs of KA treatment. Results are shown as values relative to vehicle control, which is defined as 100%. Circles represent individual lines among 5 SCLC lines used in **a** and 5 NSCLC lines (A549, H1975, HS24, LK-2, EBC-1). p = 0.0441.

(e) Immunoassays showing knock-down efficiency of indicated siRNAs in LC-KJ NSCLC cells. Shown is a representative of >3 independent experiments.

(f) Effects of knockdown of either *KYNU* or *QPRT* on NAD levels in LC-KJ cells. n = 4 (NTC/QPRT, w/ FK866) or 4 (all others) biological replicates. p < 0.0001 (NTC vs QPRT, wo FK866); p = 0.0316 (NTC vs QPRT, w/ FK866).

Data are presented as mean plus SEM. *p < 0.05, ****p < 0.0001 as determined by one-way ANOVA with a post-hoc test (**c** and **f**) or by two-tailed t-test (**d**). ns., not significant. Source data are provided as a Source Data file.

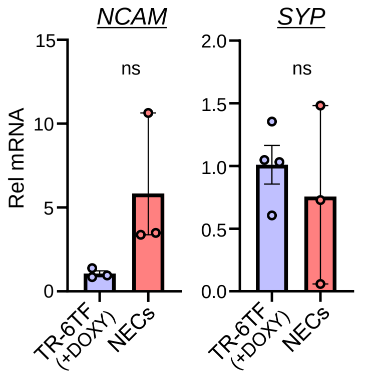

Supplementary Fig. 3. Expression of NE markers in NE-organoid and NEC lines, related to Fig. 4. Comparison of transcript levels of two NE markers between differentiated TR-6TF organoid and NEC cell lines. Each symbol represents 3 (*NCAM*) or 4 (*SYP*) biological replicates of TR-6TF and 3 NEC cell lines (SCLC 87-5, SCPC H-660 and CRPC KUCaP13). Data are presented as mean plus SEM. ns., not significant. p = 0.1187 (NCAM); p = 0.5449 (SYP). Source data are provided as a Source Data file.

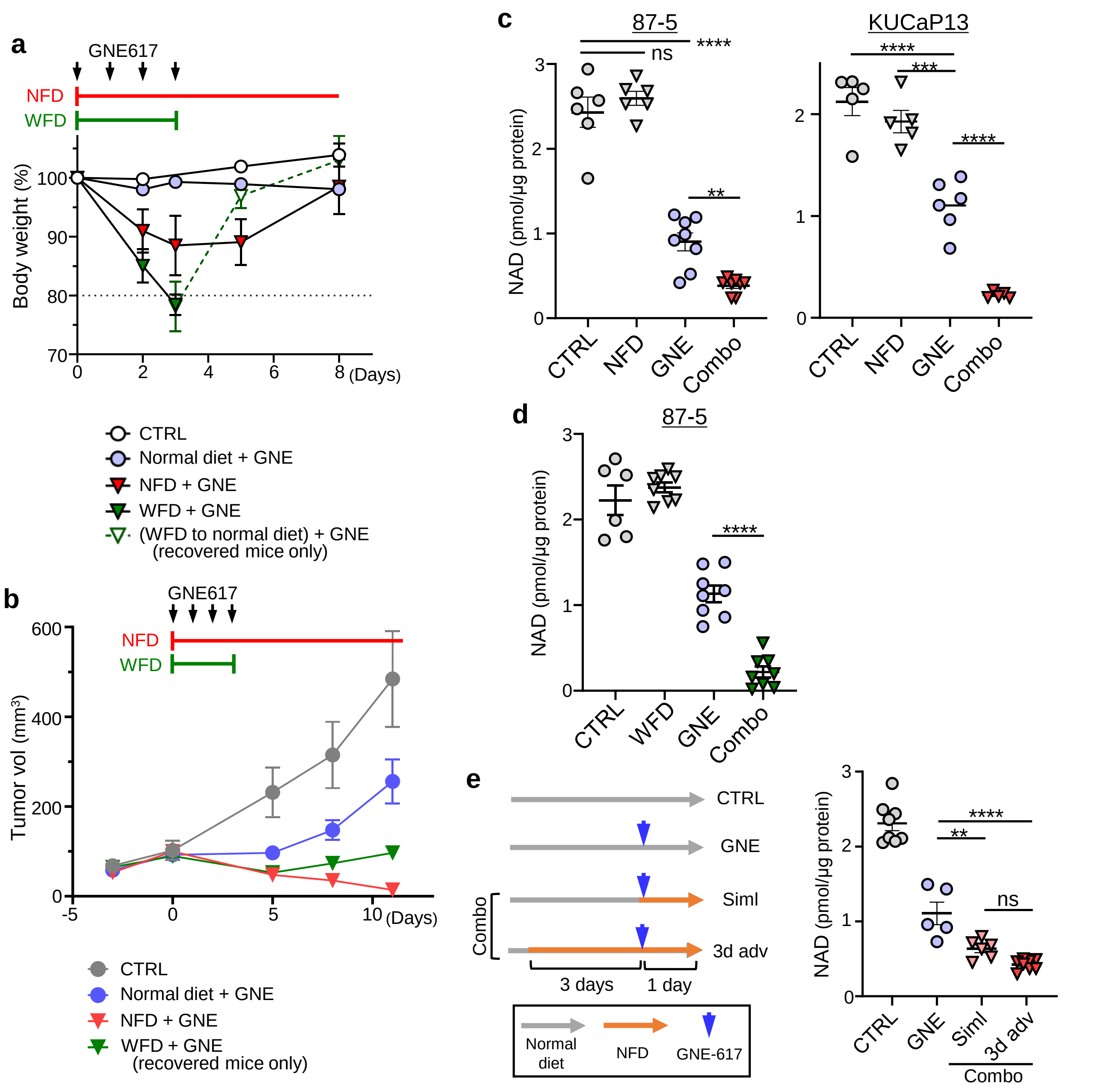

Supplementary Fig. 4. Dietary intervention affects NAMPT-targeting therapy, related to Fig. 5.

(a) Changes in body weight of mice dosed with NAMPTi (GNE, GNE-617) with or without a modifying diet. NFD, niacin-free diet; WFD, W (Trp)-free diet. Note that combining GNE with a WFD was so toxic that mice lost >20% of body weight by day 3 and were switched to a normal diet. Some but not all mice recovered body weight. Non-recovering mice were euthanized on day 4, and experiments were continued using only recovered mice. Initially, there were 3 (NFD + GNE) or 4 (all others) tumor-bearing mice, but one mouse in the WFD/GNE group was euthanized on day 4.

(b) Growth curves of Lu-139 tumors in mice receiving NAMPTi with or without modified diets, as in **a**. n = 8 (Normal diet) or 6 (NFD and WFD) tumors.

(c) NAD levels in 87-5 (left) or KUCaP13 (right) tumors in mice treated with NFD, NAMPTi (GNE) or both (Combo). n = 6 (CTRL and NFD) or 8 (GNE and Combo) 87-5 tumors; n = 5 (CTRL, NFD and Combo) or 6 (GNE) KUCaP13 tumors. $p = 0.7404$ (CTRL vs NFD, 87-5); $p < 0.0001$ (CTRL vs GNE, 87-5); $p = 0.0055$ (GNE vs Combo, 87-5); $p < 0.0001$ (CTRL vs GNE, KUCaP13); $p = 0.0001$ (NFD vs GNE, KUCaP13); $p < 0.0001$ (GNE vs Combo, KUCaP13).

(d) NAD levels in 87-5 tumors in mice treated with WFD, NAMPTi (GNE) or both (Combo). Each symbol represents a tumor. n = 6 (CTRL) or 8 (all others) tumors. $p < 0.0001$ (GNE vs Combo).

(e) Analysis of timing of NFD initiation in combined therapy. (left) Mice bearing Lu-139 tumors were treated with GNE-617, GNE-617 plus NFD or a normal diet plus vehicle only, as shown. (right) Tumors were collected one day after dosing with GNE-617 and subjected to NAD measurements. Note that effects of the NFD alone on NAD levels in Lu-139 tumors were minimal, as shown in **Fig. 5b**. n = 8 (CTRL and 3d Adv), 5 (GNE) or 6 (Siml) tumors.

$p = 0.0084$ (GNE vs Siml); $p < 0.0001$ (GNE vs 3d Adv); $p = 0.3112$ (Siml vs 3d Adv).

Data are presented as mean plus SEM. **$p < 0.01$, ***$p < 0.001$, ****$p < 0.0001$ as determined by one-way ANOVA with a post-hoc test (**c**, **d** and **e**). ns., not significant. Source data are provided as a Source Data file.

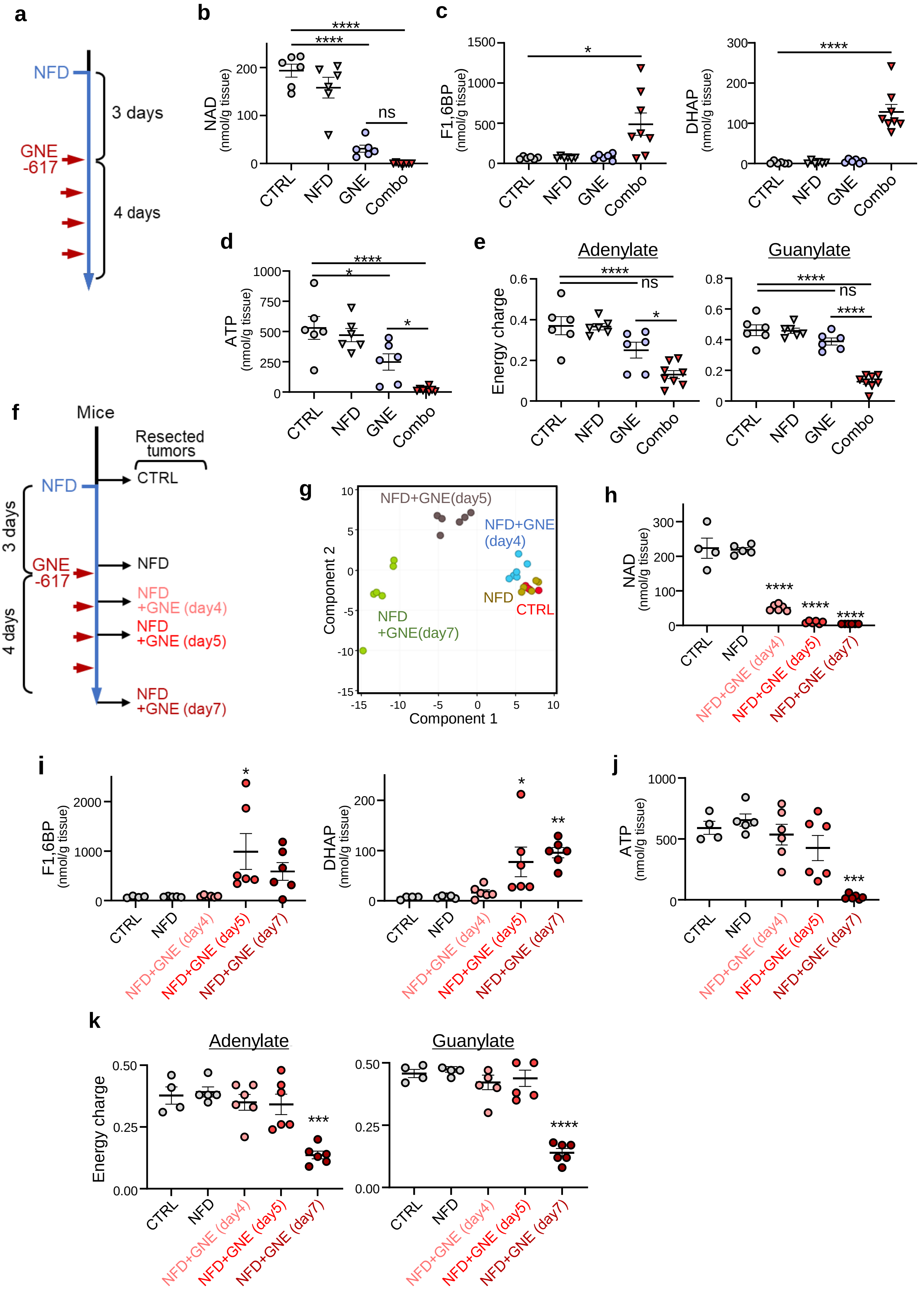
a
NFD
GNE
-617
3 days
4 days
b
NAD
(nmol/g tissue)
****
****
ns
CTRL
NFD
GNE
Combo
c
F1,6BP
(nmol/g tissue)
*
DHAP
(nmol/g tissue)
****
d
ATP
(nmol/g tissue)
****
*
*
e
Adenylate
Guanylate
Energy charge
****
ns
*
****
ns
****
f
Mice
Resected
tumors
CTRL
NFD
GNE
-617
3 days
4 days
NFD
+GNE (day4)
NFD
+GNE (day5)
NFD
+GNE (day7)
g
NFD+GNE(day5)
NFD+GNE
(day4)
NFD
CTRL
NFD
+GNE(day7)
Component 2
Component 1
h
NAD
(nmol/g tissue)
****
****
****
NFD+GNE (day4)
NFD+GNE (day5)
NFD+GNE (day7)
i
F1,6BP
(nmol/g tissue)
*
DHAP
(nmol/g tissue)
*
**
j
ATP
(nmol/g tissue)
***
k
Adenylate
Guanylate
Energy charge
***
****

Supplementary Fig. 5. Effects of niacin restriction plus NAMPT inhibition on tumor metabolism, related to Fig. 5.

(a) Shown is design of experiment performed to monitor effects of NFD, NAMPTi or a combination of both on tumor metabolism. Mice bearing Lu-139 tumors were fed a normal diet or a NFD for 3 days and then treated with GNE-617 once a day for 4 more days. Tumors were collected on day 7 and subjected to NAD measurement and metabolomic analysis (**b-e**). In **b-e**, n = 5 (CTRL, NFD and GNE) or 7 (Combo) tumors.

(b) NAD levels in tumors collected as in **a**. $p < 0.0001$ (CTRL vs GNE); $p < 0.0001$ (CTRL vs Combo); $p = 0.2847$ (GNE vs Combo).

(c) Levels of glycolytic intermediates upstream of GAPDH in indicated groups. $p = 0.0102$ (CTRL vs Combo, F1,6BP); $p < 0.0001$ (CTRL vs Combo, DHAP).

(d, e) ATP levels (**d**) and energy charge (**e**) of tumors. $p = 0.0183$ (CTRL vs GNE); $p < 0.0001$ (CTRL vs Combo); $p = 0.0473$ (GNE vs Combo) (**d**). $p = 0.0632$ (CTRL vs GNE, Adenylate); $p < 0.0001$ (CTRL vs Combo, Adenylate); $p = 0.0431$ (GNE vs Combo, Adenylate); $p = 0.1682$ (CTRL vs GNE, Guanylate); $p < 0.0001$ (CTRL vs Combo, Guanylate); $p < 0.0001$ (GNE vs Combo, Guanylate) (**e**).

(f) Design of time-course analysis of metabolic changes in tumors in mice receiving NAD-targeting therapy. Mice bearing Lu-139 tumors were fed a NFD for 3 days prior to being treated with GNE-617 once a day for 4 more days. Tumor samples were collected at days 0, 3, 4, 5 and 7, and subjected to NAD measurement and metabolomic analysis (**g-k**). In **g-k**, n = 4 (CTRL), 5 (NFD) or 6 (all others) tumors.

(g) PCA analyses of metabolomes of Lu-139 tumors in mice subjected to combined NFD plus NAMPTi therapy.

(h) NAD levels in tumors collected as in **f**. $p < 0.0001$ (day 4); $p < 0.0001$ (day 5); $p < 0.0001$ (day 7).

(i) Levels of glycolytic intermediates upstream of GAPDH in tumors collected as in **f**. $p = 0.0208$ (day 5, F1,6BP); $p = 0.0213$ (day 5, DHAP); $p = 0.0036$ (day 7, DHAP).

(j, k) ATP levels (**j**) and energy charge (**k**) of tumors collected as in **e.** $p = 0.0001$ (day 7) (**j**). $p = 0.0001$ (day 7, Adenylate); $p < 0.0001$ (day 7, Guanylate) (**k**).

Data are presented as mean plus the SEM. *p < 0.05, **p < 0.01, ***p < 0.001, ****p < 0.0001 as determined by one-way ANOVA with a post-hoc test (**b-e** and **h-k**). ns., not significant. Source data are provided as a Source Data file.

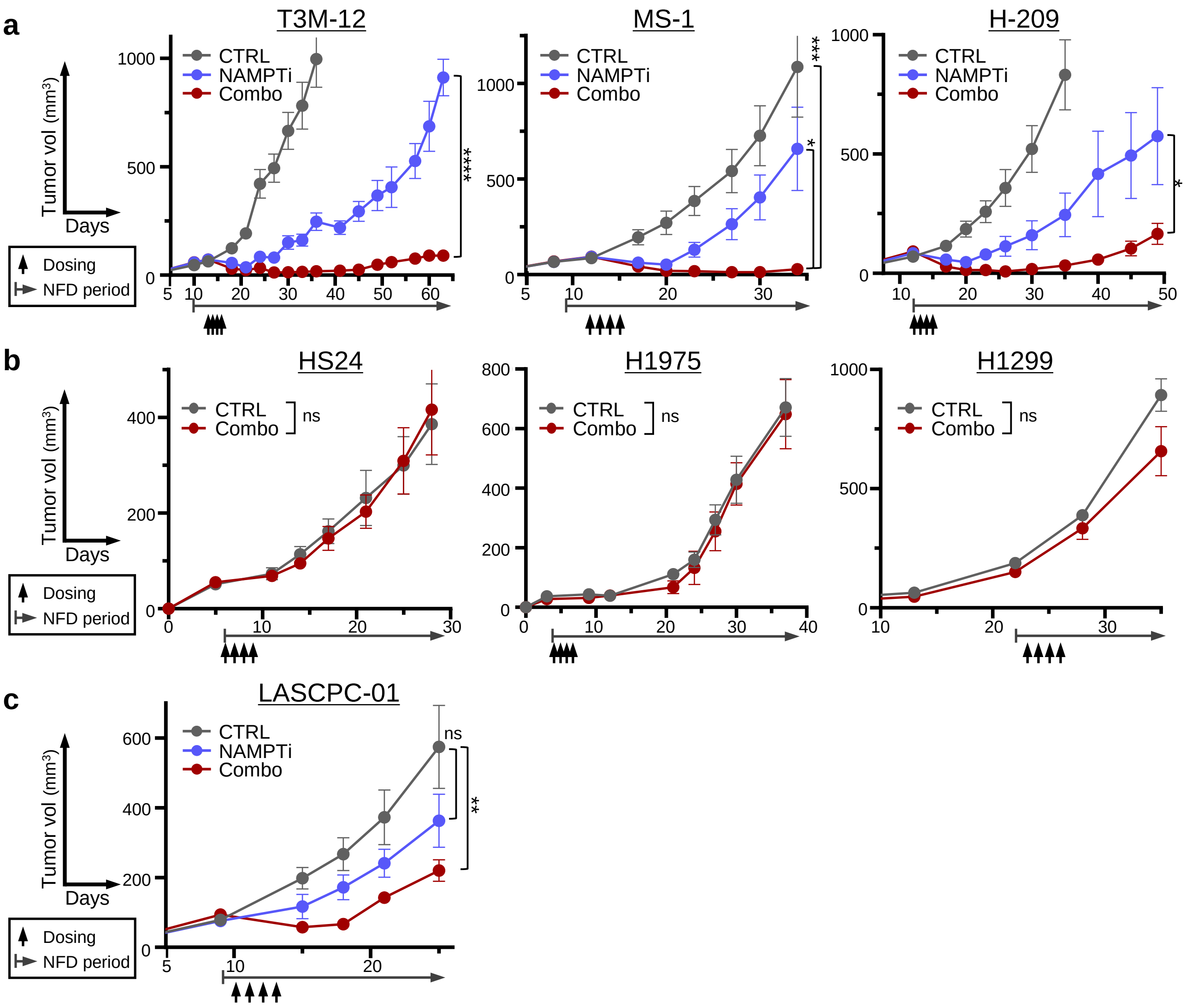

Supplementary Fig. 6. Efficacy of NAMPTi/NFD combined therapy in various tumor models, related to Fig. 5. **(a)** Growth curves of tumors derived from 3 SCLC lines (T3M-12, MS-1, and H209) in mice treated with either NFD, the NAMPTi GNE-617, or a combination of both. n = 8 (CTRL and Combo) or 6 (NAMPTi) T3M-12 tumors; n = 5 (CTRL), 7 (NAMPTi) or 10 (Combo) MS-1 tumors; n = 6 (CTRL), 8 (NAMPTi), or 10 H-209 tumors. $p < 0.0001$ (NAMPTi vs Combo, day 63, T3M-12); $p = 0.0006$ (CTRL vs Combo, day 34, MS-1); $p = 0.0180$ (NAMPTi vs Combo, day 34, MS-1); $p = 0.0440$ (NAMPTi vs Combo, day 49, H-209).

(b) Growth curves of tumors derived from 3 NSCLC lines (HS24, H1975, H1299) in mice treated either with a combination of NFD and GNE-617 (Combo) or a normal diet plus vehicle (CTRL). n = 8 (CTRL) or 6 (Combo) HS24 tumors; n = 6 (CTRL) or 4 (Combo) H1975 tumors; n = 6 (CTRL) or 8 (Combo) H1299 turmos. $p = 0.8159$ (day 28, HS24); $p = 0.8881$ (day 37, H1975); $p = 0.1034$ (day 35, H1299).

(c) Growth curves of tumors derived from the SCPC line LASCPC-01 in mice treated with NFD, GNE-617 or a combination of both. n = 6 (CTRL), 4 (NAMPTi) or 8 (Combo) tumors. $p = 0.2321$ (CTRL vs NAMPTi, day 25); $p = 0.0097$ (CTRL vs Combo, day 25).

Data are presented as mean plus SEM. *p < 0.05, **p < 0.01, ***p < 0.001, ****p < 0.0001 as determined by one-way ANOVA with a post-hoc test (**a** (MS-1) and **c**) or by two-tailed t-test (**a** (T3M-12 and H-209) and **b**). ns., not significant. Source data are provided as a Source Data file.

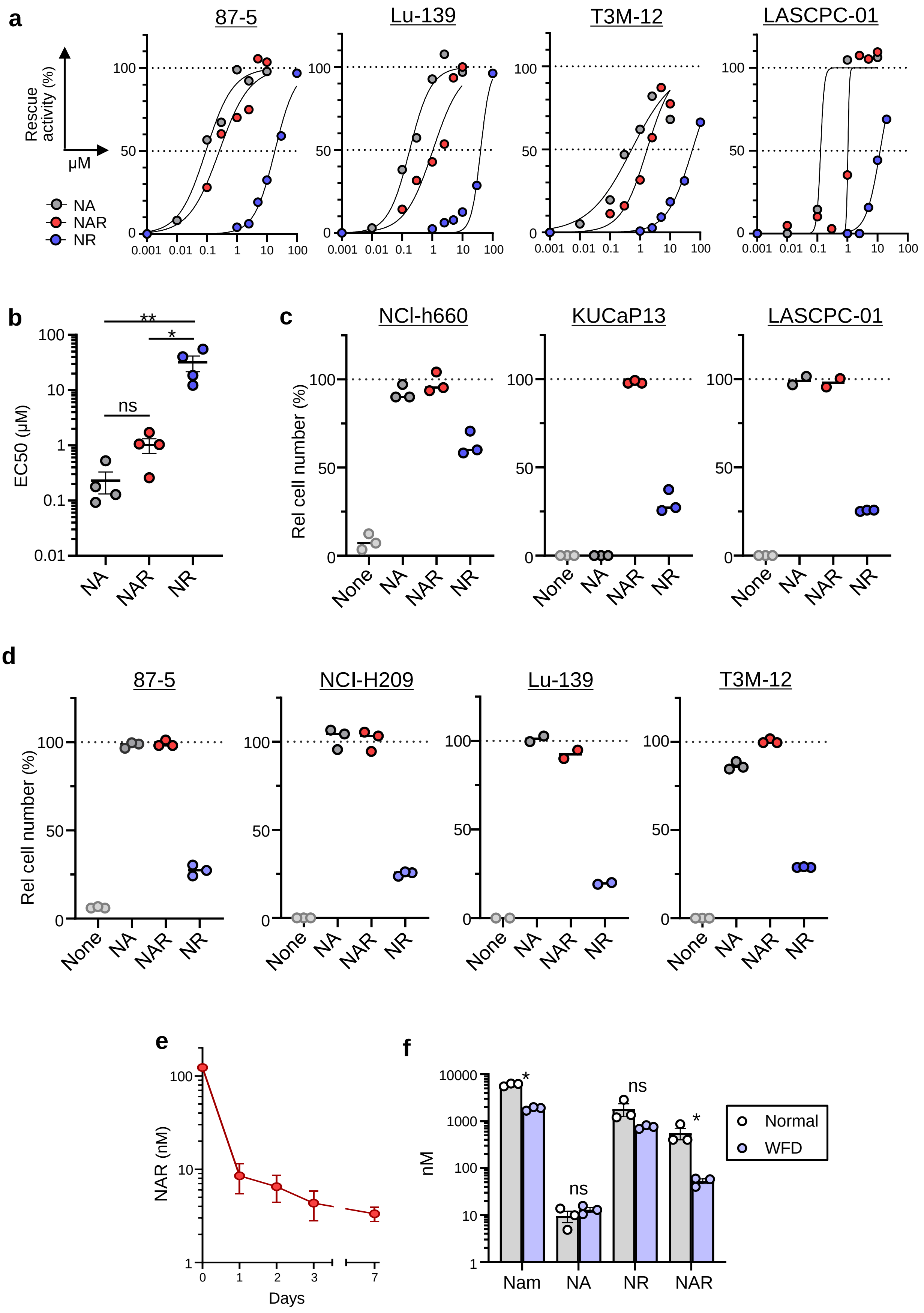

a
87-5
Lu-139
T3M-12
LASCPC-01
Rescue activity (%)
μM
NA
NAR
NR
b
EC50 (μM)
ns
**
*
c
NCI-h660
KUCaP13
LASCPC-01
Rel cell number (%)
None
d
87-5
NCI-H209
Lu-139
T3M-12
e
NAR (nM)
Days
f
nM
Normal
WFD
Nam

Supplementary Fig. 7. Effects of niacin supplementation in vitro and of NAD precursor restrictions in mice, related to Fig. 6.

(a) Rescue experiments using various concentrations of NR, NAR or NA in cells treated 4 days with 20 nM FK866. The number of viable cells was determined and rescue was quantified using the following formula: rescue activity = {(number of cells treated with FK866 plus niacin) – (number of cells treated with FK866 alone)}/{(number of untreated cells) – (number of cells treated with FK866 alone)}. Shown are representative data from 2 or 3 independent experiments.

(b) Comparison of EC_{50} values (niacin concentrations allowing 50% rescue from FK866 toxicity) in **a**. Circles represent each cell line. n = 4 cell lines. p = 0.9949 (NA vs NAR); p = 0.0097 (NA vs NR); p = 0.0112 (NAR vs NR).

(c, d) Effects of exogenous niacin on growth suppression by FK866 in NEPC (**c**) and SCLC (**d**) lines. Cells were treated 4 days with 20 nM FK866 plus either the indicated 10 mM niacin (NA, NAR or NR) or vehicle (none). Viable cells were counted and shown as values relative to untreated controls, which were defined as 100%. n = 2 (LASCPC-01) or 3 (NCI-H660 and KUCaP13) biological replicates (**c**). n = 2 (Lu-139) or 3 (all others) biological replicates (**d**).

(e) Serum NAR levels in mice treated with NFD. Day 0 defines the day diets were switched from normal to NFD. n = 3 (0, 3 and 7 hrs) or 4 (1 and 2 hrs) mice.

(f) Serum levels of niacin molecules in mice fed a normal diet or a WFD for 3 days. n = 3 mice (both groups). p = 0.0177 (Nam); p = 0.3043 (NA); p = 0.1195 (NR); p = 0.0309 (NAR).

Data are presented as mean plus SEM. *p < 0.05, **p < 0.01 as determined by one-way ANOVA with a post-hoc test (**b**) or two-tailed t-test (**f**). ns., not significant. Source data are provided as a Source Data file.

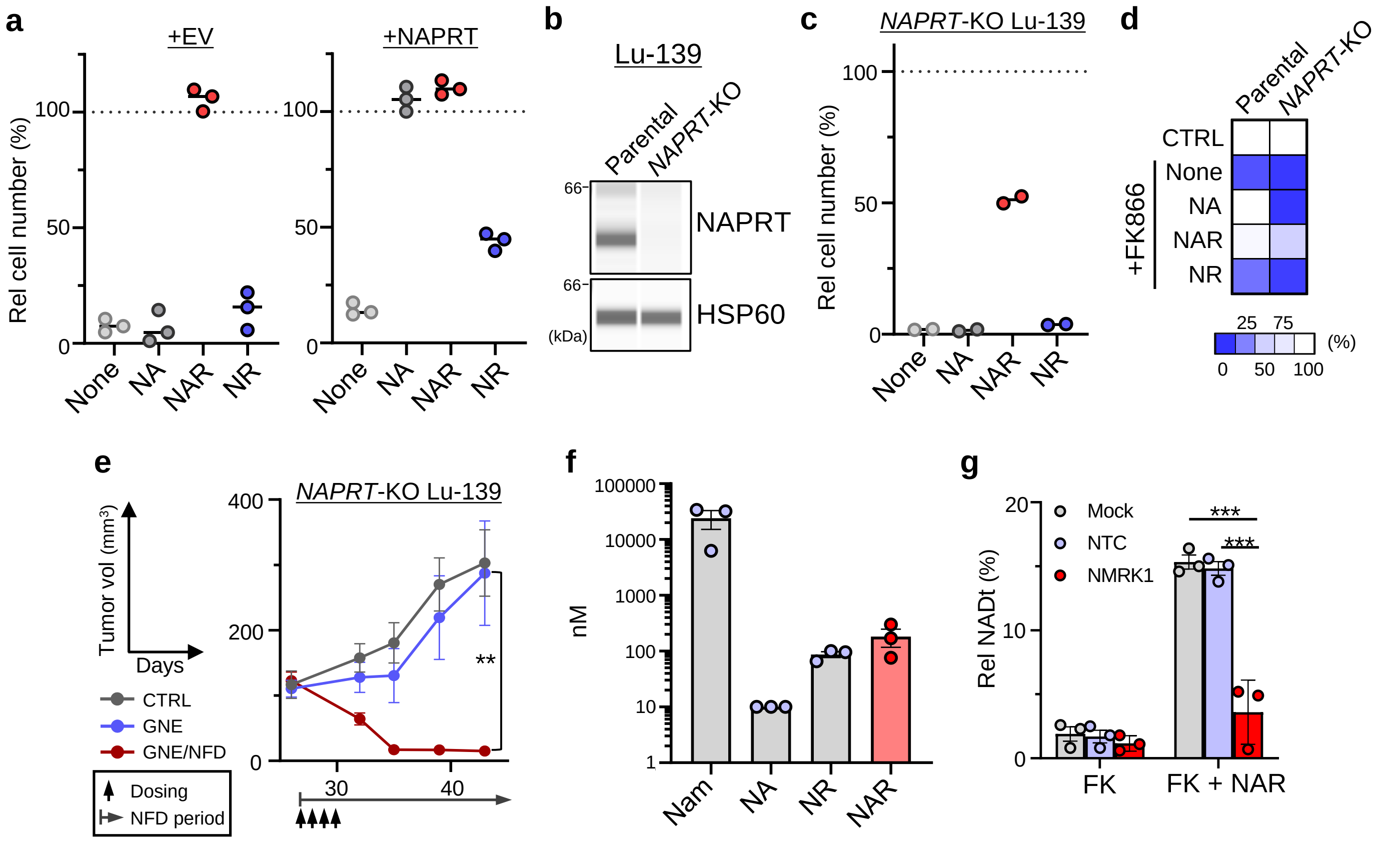

Supplementary Fig. 8. Effects of *NAPRT*-KO and *Nmrk1*-knockdown in SCLC cells, related to Fig. 6.

(a). Effects of exogenous niacin on growth suppression by FK866 in *NAPRT*-KO 87-5 clone transduced with either exogenous NAPRT cDNA or empty vector (EV). Data shown are relative to untreated control cells. n = 3 biological replicates.

(b) Immunoassays of *NAPRT*-KO Lu-139 clones. Shown is a representative of 3 independent experiments.

(c) Effects of exogenous niacin on growth suppression by FK866 in clone in **b**. Data shown are relative to untreated control cells. n = 2 biological replicates.

(d) Comparison of exogenous niacin effects on growth suppression by FK866 between parental Lu-139 cells and an *NAPRT*-KO clone.

(e) Growth curves of *NAPRT*-KO Lu-139 xenograft tumors treated as in **Fig. 6f**. n = 6 (CTRL) or 8 (GNE and GNE/NFD) tumors. $p = 0.0052$ (GNE vs GNE/NFD, day 43).

(f) Levels of serum niacin in mice fed the NFD for 3 days and then injected (ip) with NAR. Serum samples were collected 24 hr later. n = 3 mice.

(g) Effects of *NMRK1* knockdown on NAD levels. n = 3 independent experiments. FK, FK866. $p = 0.0004$ (Mock vs NMRK1, FK + NAR); $p = 0.0005$ (NTC vs NMRK1, FK + NAR).

Data are presented as mean plus the SEM. $^{**}p < 0.01$, $^{***}p < 0.001$ as determined by one-way ANOVA with a post-hoc test (e and g). Source Data are provided as a Source Data file.

a

NFD

↓ + Nam, NA NR or NAR

NamSD
NASD
NRSD
NARSD

Lactobacillus ATCC-8014 bioassay

	Niacin (μg/g diet)
Normal diet	107
NFD	< 0.03
NamSD	127
NASD	116
NRSD	49
NARSD	68

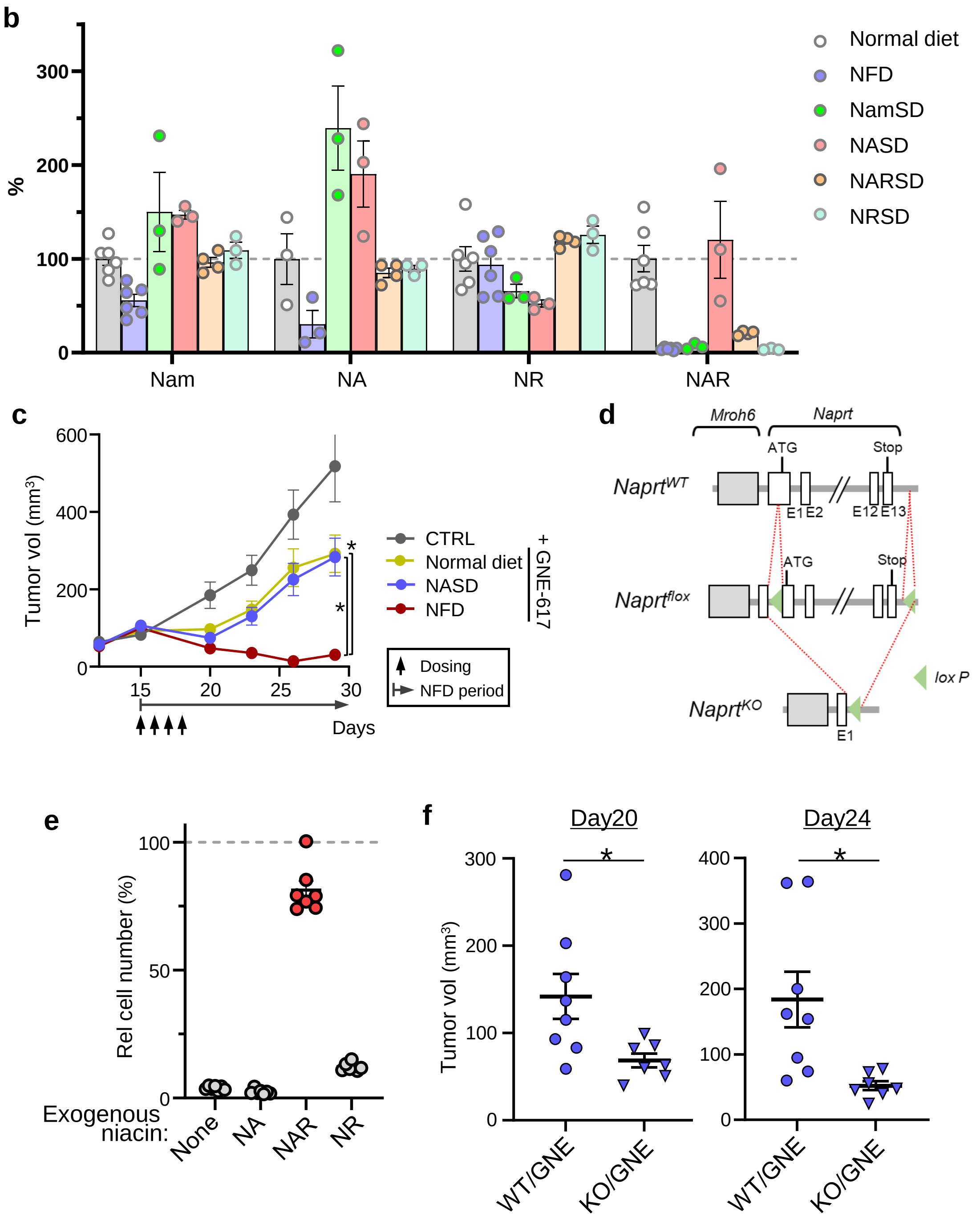

Supplementary Fig. 9. Effects of dietary niacin or *Naprt* deficiency on serum niacin levels and efficacy of NAMPT-targeting therapy, related to Fig. 7.

(**a**) Experiments analyzing effects of niacin addback to NFD. (left) Various supplemented diets (SD) were established by adding individual niacin species (Nam, NA, NR or NAR) to the NFD. (right) Those diets were tested for niacin content using *Lactobacillus* ATCC-8014. Data are averages of two technical replicates.

(**b**) Effects of niacin SDs shown in (a) on serum levels of niacin molecules in mice. NFD served as the control. n = 6 (Normal diet and NFD) or 3 (all others) mice.

(**c**) Growth curve of Lu-139 tumors in mice treated with NAMPTi (GNE-617) plus either NFD or a NA-supplemented NFD (NASD). n = 7 (CTRL), 8 (Normal diet w/ GNE-617) or 6 (NASD and NFD, GNE-617) tumors. p = 0.0240 (Normal diet vs NFD, GNE-617); p = 0.0462 (NASD vs NFD, GNE-617).

(**d**) Generation of *Naprt*-KO mice. *Naprt*-floxed mice were crossed with CAG-Cre-transgenic mice to delete the floxed region. Boxes represent exons.

(**e**) Effects of FK866 on A2780 cell growth and potential rescue by niacin. Cells were treated 4 days with 20 nM FK866 in the presence or absence of exogenous 10 mM NA, NAR or NR, or left untreated. Cells were counted and shown as values relative to untreated controls, which were defined as 100%. n = 6 biological replicates.

(**f**) Volume of A2780 tumors on days indicated in mice treated with NAMPTi as in **Fig 7g**. n = 7 (WT/GNE) or 8 (KO/GNE) tumors. p = 0.0238 (day 20); p = 0.0136 (day 24).

Data are presented as mean plus SEM. *$p < 0.05$ as determined by one-way ANOVA with a post-hoc test (**c**) or by two-tailed t-test (**f**). ns., not significant. Source data are provided as a Source Data file.
