# Supplementary material for: Niacin restriction with NAMPT-inhibition is synthetic lethal to neuroendocrine carcinoma
Source: Nat Commun. 2023 Dec 13;14:8095. doi: 10.1038/s41467-023-43630-3 (PMC10719245; doi:10.1038/s41467-023-43630-3)
Supplement: Supplementary file 3 — Description of Additional Supplementary Files [file 41467_2023_43630_MOESM3_ESM.pdf]

Description of Additional Supplementary Files

Supplementary Data 1
Description: qRT-PCR analyses were performed using both sense and anti-sense primers in combination with TaqMan probes from a universal probe library (UPL) obtained from Roche. Alternatively, assays were done using primers and TaqMan probe provided in the TaqMan Gene Expression Assay kit from Thermo Fisher.
