# Supplementary material for: Niacin restriction with NAMPT-inhibition is synthetic lethal to neuroendocrine carcinoma
Source: Nat Commun. 2023 Dec 13;14:8095. doi: 10.1038/s41467-023-43630-3 (PMC10719245; doi:10.1038/s41467-023-43630-3)
Supplement: Supplementary file 5 — Reporting Summary [file 41467_2023_43630_MOESM5_ESM.pdf]

nature portfolio

Corresponding author(s): Nobuhiro Tanuma

Last updated by author(s): Oct 12, 2023

Reporting Summary

Nature Portfolio wishes to improve the reproducibility of the work that we publish. This form provides structure for consistency and transparency in reporting. For further information on Nature Portfolio policies, see our Editorial Policies and the Editorial Policy Checklist.

Statistics

For all statistical analyses, confirm that the following items are present in the figure legend, table legend, main text, or Methods section.

n/a	Confirmed	
[ ]	[x]	The exact sample size (*n*) for each experimental group/condition, given as a discrete number and unit of measurement
[ ]	[x]	A statement on whether measurements were taken from distinct samples or whether the same sample was measured repeatedly
[ ]	[x]	The statistical test(s) used AND whether they are one- or two-sided<br>*Only common tests should be described solely by name; describe more complex techniques in the Methods section.*
[x]	[ ]	A description of all covariates tested
[x]	[ ]	A description of any assumptions or corrections, such as tests of normality and adjustment for multiple comparisons
[ ]	[x]	A full description of the statistical parameters including central tendency (e.g. means) or other basic estimates (e.g. regression coefficient) AND variation (e.g. standard deviation) or associated estimates of uncertainty (e.g. confidence intervals)
[ ]	[x]	For null hypothesis testing, the test statistic (e.g. *F*, *t*, *r*) with confidence intervals, effect sizes, degrees of freedom and *P* value noted<br>*Give P values as exact values whenever suitable.*
[x]	[ ]	For Bayesian analysis, information on the choice of priors and Markov chain Monte Carlo settings
[x]	[ ]	For hierarchical and complex designs, identification of the appropriate level for tests and full reporting of outcomes
[x]	[ ]	Estimates of effect sizes (e.g. Cohen's *d*, Pearson's *r*), indicating how they were calculated

Our web collection on statistics for biologists contains articles on many of the points above.

Software and code

Policy information about availability of computer code

Data collection	Compass for SW, version 6.1.0 (Protein Simple).<br>Evolution-Capt Edge (Vilber Lourmat).<br>Gen 5, version 3.2.1.0 (Bio-Tek Instruments).<br>LightCycler 480 SW User Defined Workflow for cobas z 480, version 1.5.1.62 (Roche).<br>MassHunter Workstaion-Data Acquisition, version B.05.00 (Agilent).
Data analysis	Compass for SW, version 6.1.0 (Protein Simple).<br>MassHunter Workstaion-Quantitative Analysis, version B.05.00 (Agilent).<br>GraphPad Prism, version 9.0.2 (GraphPad Software)

For manuscripts utilizing custom algorithms or software that are central to the research but not yet described in published literature, software must be made available to editors and reviewers. We strongly encourage code deposition in a community repository (e.g. GitHub). See the Nature Portfolio guidelines for submitting code & software for further information.

Data

Policy information about availability of data

All manuscripts must include a data availability statement. This statement should provide the following information, where applicable:

- Accession codes, unique identifiers, or web links for publicly available datasets
- A description of any restrictions on data availability
- For clinical datasets or third party data, please ensure that the statement adheres to our policy

The metabolome data generated in this study have been deposited in the "MetaboLights" database under accession codes MTBLS7251, MTBLS7252 and MTBLS7253 [https://www.ebi.ac.uk/metabolights/]. The CRISPR-KO and mRNA expression data used in this study are available in the "DepMap portal" [https://depmap.org/portal/]. Source data are provided with this paper.

Research involving human participants, their data, or biological material

Policy information about studies with human participants or human data. See also policy information about sex, gender (identity/presentation), and sexual orientation and race, ethnicity and racism.

Reporting on sex and gender	The organoid TR-6TF was established by genetic manipulation of an organoid line established from non-tumor colon tissue from a 77-year-old female colon cancer patient, as described in a previous study (Kawasaki et al, Cell '20). These results are not expected to be affected by gender.
Reporting on race, ethnicity, or other socially relevant groupings	No analysis of race, ethnicity, or other socially-relevant groups is reported here.
Population characteristics	As noted above, a 77-year-old woman with colon cancer.
Recruitment	Patient recruitment was described in a previous study reporting organoid establishment (Kawasaki et al, Cell '20).
Ethics oversight	The ethics committees at Miyagi Cancer Center and Ethical committees of Keio University School of Medicine approved the study.

Note that full information on the approval of the study protocol must also be provided in the manuscript.

Field-specific reporting

Please select the one below that is the best fit for your research. If you are not sure, read the appropriate sections before making your selection.

☒ Life sciences ☐ Behavioural & social sciences ☐ Ecological, evolutionary & environmental sciences

For a reference copy of the document with all sections, see nature.com/documents/nr-reporting-summary-flat.pdf

Life sciences study design

All studies must disclose on these points even when the disclosure is negative.

Sample size	No statistical methods were used to predetermine sample size. Sample size for mouse experiments was determined from our pilot data, anticipated biological variables, and the literature (Okasho et al., Cancer Sci 2021 [https://doi.org/10.1111/cas.14935], Park et al., Science 2018 [https://doi.org/10.1126/science.aat5749], Chalishazar et al., Clin Cancer Res 2019 [https://doi.org/10.1158/1078-0432.CCR-18-4140]). Accordingly, 2-7 mice were used per group. Sample size for other in vitro experiments was chosen based on standards in the field. Those experiments were performed using at least two technical replicates. Each experiment was repeated independently at least three times. Sample sizes are reported in corresponding figure legends.
Data exclusions	No data were excluded in the study.
Replication	MS analyses in Fig. 7a and niacin tests in Supplementary Fig. 9a were evaluations of rodent diets obtained from manufacturers, and each was performed once.<br>qRT-PCR analyses shown in Figs 4g and 6h were also performed once because 1) their purpose was to compare groups of multiple cell lines rather than to compare individual cell lines with each other, and 2) cell lines analyzed were not subjected to treatment and were thus anticipated to show little variation.<br>All other experiments were performed at least twice with comparable results.
Randomization	Mice were randomized before intervention for allocation into experimental groups. No formal randomization method was used. For cell culture experiments, random plates/dishes of cells were chosen for treatment.
Blinding	Experiments were not blinded as investigators needed to distinguish controls from other groups.

Reporting for specific materials, systems and methods

We require information from authors about some types of materials, experimental systems and methods used in many studies. Here, indicate whether each material, system or method listed is relevant to your study. If you are not sure if a list item applies to your research, read the appropriate section before selecting a response.

Materials & experimental systems

n/a	Involved in the study
☐	☒ Antibodies
☐	☒ Eukaryotic cell lines
☒	☐ Palaeontology and archaeology
☐	☒ Animals and other organisms
☒	☐ Clinical data
☒	☐ Dual use research of concern
☒	☐ Plants

Methods

n/a	Involved in the study
☒	☐ ChIP-seq
☒	☐ Flow cytometry
☒	☐ MRI-based neuroimaging

Antibodies

Antibodies used

Anti-PAR mAb (10407, IBL, 1:200), anti-Actin mAb (A3853, Thermo Fisher, 1:500), anti-NAMPT (ab45890, abcam, 1:500), anti-NAPRT (ab127699, abcam, 1:2000), anti-NADSYN1 (ab171942, abcam, 1:900), anti-QPRT (ab171944, abcam, 1:1000), anti-HSP60 (AF1800, ProteinSimple, 1:500), anti-KYNU (11796-1-AP, ProteinTech, 1:200), anti-HAAO (12791-1-AP, ProteinTech, 1:300). anti-mouse IgG-HRP (sc-2055, SantaCruz, 1:3000), anti-rabbit IgG-HRP (5220-0336, SeraCare, 1:3000), anti-mouse Ig-HRP (042-205, Protein Simple, no dilution), anti-rabbit Ig-HRP (042-206, Protein Simple, no dilution) and anti-mouse Ig-NIR (043-821, Protein Simple, 1:200).

Validation

Antibodies were validated based on the size of band in Western blotting or immunoassays (molecular weight), specificity/selectivity assessed by using samples from knock-down/knock-out/over-expressing/inhibitor-treated cells, and reproducibility of the results.
- Anti-PAR: https://www.ibl-japan.co.jp/search/product/detail/id=3588
- Anti-Actin: https://www.citeab.com/antibodies/2288234-a3853-anti-actin-antibody-mouse-monoclonal
- Anti-NAMPT: https://www.abcam.com/visfatin-antibody-ab45890.html.
- Anti-NAPRT: https://www.abcam.co.jp/products/primary-antibodies/naprt1-antibody-ab127699.html
- Anti-NADSYN1: https://www.abcam.co.jp/nadsyn1-antibody-epr10611-ab171942.html
- Anti-QPRT: https://www.abcam.co.jp/qprt-antibody-epr11941b-ab171944.html
- Anti-HSP60: https://www.rndsystems.com/products/human-mouse-rat-hsp60-antibody_af1800
- Anti-KYNU: https://www.ptglab.co.jp/products/KYNU-Antibody-11796-1-AP.htm
- Anti-HAAO: https://www.ptglab.co.jp/products/HAAO-Antibody-12791-1-AP.htm

Eukaryotic cell lines

Policy information about cell lines and Sex and Gender in Research

Cell line source(s)

Lu-134, Lu-139, Lu-165, T3M-12, 87-5, MS-1, S1, A549, 293T, LC-KJ, EBC-1, LK-2, DU145, LNCaP, MOLM-14, NOMO-1, THP-1 and U937 were obtained from Riken BRC.
H1299, H1975, VCaP, 22Rv1, PC-3, NCI-H660, LASCPC-01, PRE-1 and NCI-H209 were obtained from ATCC.
IMR-90 and TIG3 were obtained from JCRB.
HS24: from Dr Maemondo's Lab (Maemondo et al, Cancer Res '04).
A2780: from Dr Carla Grandori's Lab (Kudo et al, FEBS Lett '20).
KUCaP13: was previously established in the Akamatsu Lab from tumor tissue from a male prostate cancer patient (Okasyo et al, Cancer Sci '21).
MEF-KRAS and LE-KRAS: was previously established in the Tanuma Lab by immortalization/transformation of cells isolated from female mice (Morita et al, Cancer Cell '18).

Authentication

Cell line authentication test were not performed.

Mycoplasma contamination

All lines were verified as mycoplasma-free using a MycoAlert kit (Lonza).

Commonly misidentified lines (See ICLAC register)

No commonly misidentified cell lines were used in the study.

Animals and other research organisms

Policy information about studies involving animals; ARRIVE guidelines recommended for reporting animal research, and Sex and Gender in Research

Laboratory animals

Mouse strains used and corresponding suppliers/sources are as follows:

NOG (NOD.Cg-Prkdcscid Il2rg^tm1Sug/Jic) mice: In-Vivo Science (Tokyo, Japan).
Rag1-KO (B6.129S7-Rag1^tm1Mom/J) mice: The Jackson Laboratory (Bar Harbor, ME).
CAG-Cre (B6.Cg-Tg(CAG-Cre)CZ-MO2Osb) mice: RikenBRC (Tsukuba, Japan).
Narprt-flox (C57BL/6-Naprt^tm1Ntan) mice: Tanuma Laboratory.
Mice were maintained in specific pathogen-free (SPF) facilities with a 12-hour light-dark cycle, with controlled temperature ~25ºC and controlled humidity ~50%.
All NOG mice and Naprt;Rag1 double KO mice used in this study were 8 to 12 week-old females. Mice used in Figs 7e and f were 8 to 12 week-old male Naprt-KO males. Naprt-KO mice were the offspring of Naprt-flox and CAG-Cre strains.

Wild animals	No wild animals were used in the study.
Reporting on sex	For transplantation experiments, only female mice were used to minimize animal fighting after randomization. In experiments reported in Figs 7e and f, only male mice were used. Results reported here are not expected to be affected by gender. In pilot experiments, blood niacin levels and liver NAPRT protein levels were comparable in male and female mice.
Field-collected samples	No field collected samples were used in the study.
Ethics oversight	All animal experiments were performed with approval of the Miyagi Cancer Center Research Institute Animal Care and Use Committee.

Note that full information on the approval of the study protocol must also be provided in the manuscript.
